# Blood Biomarkers Predict Cardiac Workload Using Machine Learning

**DOI:** 10.1155/2021/6172815

**Published:** 2021-06-01

**Authors:** Lan Shou, Wendy Wenyu Huang, Andrew Barszczyk, Si Jia Wu, Helen Han, Alex Waese-Perlman, Lulu Chen, Jing Wei, Hong Luo, Kang Lee

**Affiliations:** ^1^The Affiliated Hospital of Hangzhou Normal University, Hangzhou Normal University, 58 Haishu Rd., Hangzhou, Zhejiang, China 311121; ^2^Applied Psychology and Human Development, University of Toronto, 252 Bloor St. West, Toronto, Ontario, Canada M5S 1V6; ^3^Department of Physiology, University of Toronto, Medical Sciences Building, Rm. 3306. 1 King's College, Toronto, Ontario, Canada M5S 1A8

## Abstract

**Introduction:**

Rate pressure product (the product of heart rate and systolic blood pressure) is a measure of cardiac workload. Resting rate pressure product (rRPP) varies from one individual to the next, but its biochemical/cellular phenotype remains unknown. This study determined the degree to which an individual's biochemical/cellular profile as characterized by a standard blood panel is predictive of rRPP, as well the importance of each blood biomarker in this prediction.

**Methods:**

We included data from 55,730 participants in this study with complete rRPP measurements and concurrently collected blood panel information from the Health Management Centre at the Affiliated Hospital of Hangzhou Normal University. We used the XGBoost machine learning algorithm to train a tree-based model and then assessed its accuracy on an independent portion of the dataset and then compared its performance against a standard linear regression technique. We further determined the predictive importance of each feature in the blood panel.

**Results:**

We found a fair positive correlation (Pearson *r*) of 0.377 (95% CI: 0.375-0.378) between observed rRPP and rRPP predicted from blood biomarkers. By comparison, the performance for standard linear regression was 0.352 (95% CI: 0.351-0.354). The top three predictors in this model were glucose concentration, total protein concentration, and neutrophil count. *Discussion*/

**Conclusion:**

Blood biomarkers predict resting RPP when modeled in combination with one another; such models are valuable for studying the complex interrelations between resting cardiac workload and one's biochemical/cellular phenotype.

## 1. Introduction

The heart continually adjusts the amount of work it does to pump blood throughout the body. This “cardiac workload” is elegantly captured by an index called the rate pressure product (RPP) [1–4], which is the product of heart rate (HR) and systolic blood pressure (SBP) (Equation (1)). Given the circadian nature of blood pressure and heart rate, resting RPP (rRPP) exhibits a strong circadian pattern; it reaches its maximum shortly after waking and remains fairly constant throughout waking hours [5]. However, daytime rRPP varies from one individual to the next. We know that robust increases in RPP caused by exercise are associated with local biochemical changes in the heart [1, 6–8] and elsewhere in the body [9]. However, it has yet to be determined the degree to which rRPP is associated with an individual's biochemical and cellular profile as reflected in the bloodstream, and which aspects of this profile are most strongly related to rRPP. Such knowledge would contribute to our understanding of human physiology and pathophysiology.

Large clinical datasets and advanced machine learning algorithms may finally make it possible to model and study such complex, multifactorial relations. Large datasets provide adequate statistical power for identifying subtle relations among variables. Further, using routinely collected clinical data ensures that models will generalize well to the general population. Advanced machine learning techniques are useful because they can efficiently process large amounts of data and model subtle, nonlinear relations between numerous predictors without explicit programming [10].

This study will investigate the degree to which an individual's rRPP is associated with their biochemical/cellular profile overall, as well as how well specific blood biomarkers explain differences in rRPP. We will do this by modeling the complex relations between a comprehensive panel of blood biomarkers and rRPP using an advanced machine learning algorithm and then quantify the prediction accuracy of this model on an independent portion of the dataset. We will then compare the performance of this model against standard linear regression to determine whether the machine learning model provides an advantage. We will further calculate the predictive importance of each blood biomarker in the model to determine which blood biomarkers best explain differences in rRPP.

## 2. Materials and Methods

### 2.1. Study Participants

Data for the present study was obtained from adults 18 years of age and older who underwent a physical examination at the Health Management Centre at the Affiliated Hospital of Hangzhou Normal University. We included 55,730 unique adults (44% female; mean age ± SD: 46 ± 13.8 years) with complete data for analysis. The use of human data in this study was approved by Research Ethics Review Committee at the Affiliated Hospital of Hangzhou Normal University, and subjects provided written informed consent to have their data used in the study.

### 2.2. Data Collection

A medical professional measured each participant's resting pulse rate and systolic blood pressure by auscultation using a stethoscope and sphygmomanometer. Each was taken as the average of 3 measurements. Pulse (bpm) and systolic blood pressure (mmHg) were multiplied together to calculate rRPP (Equation (1)).

(1) $$\begin{array}{c} \text{Resting Rate Pressure Product }\left(\text{rRPP}\right)=\text{Pulse}\times \text{Systolic Blood Pressure}. \end{array}$$

Blood samples were then drawn into sample tubes and sent to certified lab technicians at the hospital for the analysis of 29 blood items: total protein concentration (g/L), albumin concentration (g/L), globulin concentration (g/L), albumin-globulin ratio, creatinine concentration (*μ*mol/L), uric acid concentration (*μ*mol/L), white blood cell count (10^9^/L), total cholesterol concentration (mmol/L), glucose concentration (mmol/L), neutrophil percentage (% of white blood cell count), lymphocyte percentage (% of white blood cell count), monocyte percentage (% of white blood cell count), eosinophil percentage (% of white blood cell count), basophil percentage (% of white blood cell count), absolute neutrophil count (10^9^/L), absolute lymphocyte count (10^9^/L), absolute monocyte count (10^9^/L), absolute eosinophil count (10^9^/L), absolute basophil count (10^9^/L), red blood cell count (10^12^/L), hemoglobin concentration (g/L), mean red blood cell volume (fl), mean corpuscular hemoglobin (hemoglobin concentration (g/L) divided by red blood cell count (10^12^/L), expressed in picograms), mean corpuscular hemoglobin concentration (hemoglobin concentration (g/L) divided by hematocrit and expressed in g/L), red cell volume distribution width (distribution of individual red blood cell volumes, %), platelet count (10^9^/L), mean platelet volume (10^9^/L), platelet percentage (by blood volume), and platelet distribution width (distribution of individual platelet volumes, %).

### 2.3. Model Training and Validation

We prepared the data by converting each variable to a *z*-score; this standardization step allowed us to identify and subsequently remove outliers from the data (defined as 3 standard deviations above or below the mean). Next, we randomly allocated 80% of the data for training, 10% for testing, and 10% as a holdout set for validation.

We then proceeded to train a tree-based computational model and standard linear regression model to predict rRPP from the full set of blood biomarkers: total protein concentration, albumin concentration, globulin concentration, albumin-globulin ratio, creatinine concentration, uric acid concentration, white blood cell count, total cholesterol concentration, blood glucose concentration, neutrophil percentage, lymphocyte percentage, monocyte percentage, eosinophil percentage, basophil percentage, absolute neutrophil count, absolute lymphocyte count, absolute monocyte count, absolute eosinophil count, absolute basophil count, red blood cell count, hemoglobin concentration, mean red blood cell volume, mean corpuscular hemoglobin, mean corpuscular hemoglobin concentration, red cell volume distribution width, platelet count, mean platelet volume, platelet percentage, and platelet distribution width. We did so using the XGBoost machine learning algorithm and standard linear regression implemented in the Python programming language. The training and testing portions of the dataset were used to train the model.

Model accuracy was then calculated as explained variance (*R*^2^; coefficient of determination) on the independent “holdout” portion of the dataset. The importance of each feature was calculated internally by the XGBoost algorithm as a function of the number of times that variable was selected for splitting by the XGBoost algorithm and the squared improvement to the model based on that split, with the resulting value averaged across all trees in the model to arrive at the importance [11]. This value was normalized against the best-performing feature to obtain a relative importance (%) for each feature. We further investigated the ability of the top feature to predict rRPP on its own using linear regression and LOESS regression.

This entire process (beginning with random participant allocation into training, testing, and holdout portions of the dataset) was repeated 100 times to generate statistical estimates of model performance and normalized feature importance. An overall Pearson correlation was calculated by taking the square root of the mean explained variance and its 95% confidence interval across all model iterations. We also calculated the mean normalized importance and its 95% confidence interval for each feature across all iterations of the model. To visualize model performance across a range of reference rRPP values, we chose one iteration at random and plotted a scatter plot of reference versus predicted rRPP values.

## 3. Results

### 3.1. Model Performance

**Figure 1:**
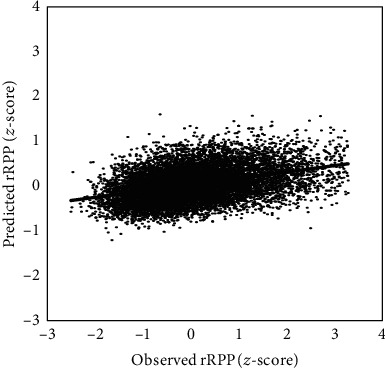
Scatterplot of observed rRPP (*z*-score) versus predicted rRPP (*z*-score). Observed rRPP was calculated from measured pulse and blood pressure. This iteration was chosen at random (out of 100 model iterations) to visualize prediction performance over a range of observed rRPPs. Line represents linear line of best fit.

We found a fair positive correlation of 0.377 (95% CI: 0.375-0.378; Pearson *r*) between observed rRPP (calculated from measured heart rate and systolic blood pressure) and rRPP predicted from blood biomarkers across 100 model iterations. This was an improvement over the linear regression model, which had a correlation of 0.352 (95% CI: 0.351-0.354). A scatter plot depicting XGBoost model performance across a range of reference rRPP values for one model iteration taken at random is depicted in Figure 1.

### 3.2. Feature Importance

**Figure 2:**
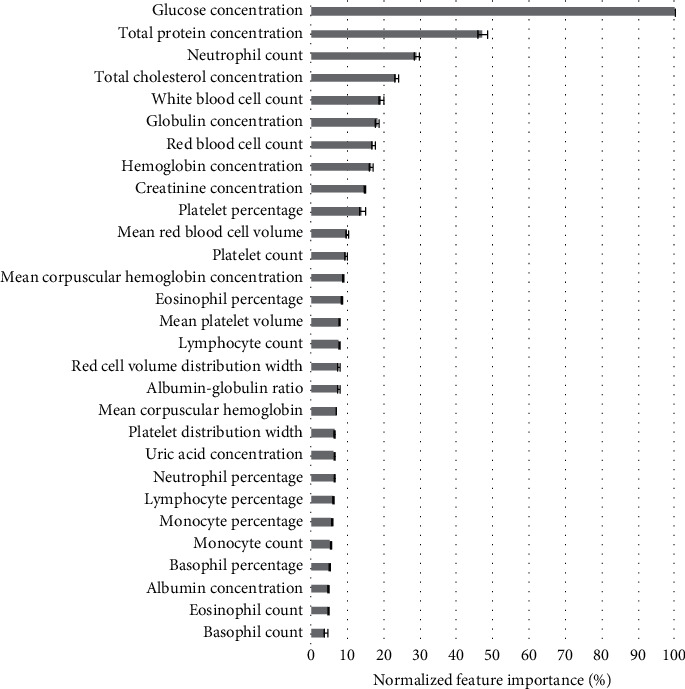
Feature importance in rRPP prediction model. Importance of each *z*-scored feature was normalized against the importance of the most important feature to provide a measure of relative importance (%).

On average across 100 iterations, glucose concentration was the most important predictor (Figure 2). Other important predictors (e.g., those with 20% normalized importance or greater) included total protein concentration (mean ± 95% CI: 47.0 ± 1.2%), neutrophil count (29.0 ± 0.7%), and total cholesterol concentration (23.4% ± 0.6%).

Given that glucose concentration dominated this model, we determined the degree to which glucose alone predicts RPP using both linear (linear regression) and a nonlinear (LOESS regression) modeling. We found that the Pearson *r* was 0.247 (95% CI: 0.245-0.249) in the linear model and 0.245 (95% CI: 0.244-0.247) in the nonlinear model. Performance was essentially equivalent with these two approaches and inferior to the performance of the XGBoost model with all predictors. Thus, the additional features above and beyond glucose explain a lot of additional variance.

## 4. Discussion/Conclusion

In this study we determined that an individual's biochemical/cellular profile is indeed a fair predictor of rRPP and that XGBoost-based models are superior to standard linear regression in modeling such relations. Within this profile, we identified several blood biomarkers that best predicted an individual's rRPP. The most important by far was glucose concentration; other important blood biomarkers (e.g., those with 20% normalized importance or greater) included total protein concentration, neutrophil count, and total cholesterol concentration. While blood glucose was highly important on its own, correlation was greatly improved when the full set of predictors was considered.

To the best of our knowledge, our study was the first to simultaneously model the relations between a comprehensive panel of blood biomarkers and rRPP. Our combinatorial machine learning-based modeling approach allowed for the potential discovery and inclusion of subtle and yet-unknown nonlinear interactions between two or more variables in the model; the explicit discovery and inclusion of such interactions among numerous predictors without the automaticity of machine learning would have been impractical.

Our finding that blood glucose was a major predictor of rRPP agrees with past studies showing the association of blood glucose with heart rate [12] and blood pressure individually. This effect is likely explained by the impact of blood sugar on the autonomic nervous system [13]. While this association with blood pressure has been demonstrated across all blood pressure quantiles in men, in women, it has only been detected in upper blood pressure quantiles (likely due to the mitigating effect of estrogen on insulin resistance) [13, 14]. We expect that our model considered this nonlinear relation and gender-based interaction in predicting rRPP. The link between blood glucose and resting cardiac workload (rRPP) is important because elevated blood glucose (e.g., in diabetics and prediabetics) is a risk factor for coronary artery disease (CAD) [15, 16]. Prolonged elevated blood glucose could trigger proatherogenic conditions of the vessels, leading to endothelial dysfunction, oxidative stress [17], increased vascular inflammation, vascular adhesion to monocytes/macrophages [18], vascular permeability [19], and secretion of prothrombotic factor (plasminogen activator inhibitor-1) [20]. This may in turn result in a higher cardiac workload.

Our finding that hemoglobin was also a fair predictor of rRPP is consistent with the fact that hemoglobin production is upregulated under conditions of reduced oxygen delivery to tissues (like rRPP). Continued poor oxygen delivery can lead to additional compensatory processes such as arterial remodeling (resulting in thickening of the myocardium) and myocardial cell death [21]. In fact, elevated hemoglobin has been associated with cardiovascular diseases [22]. Gaining a better understanding about the relations between various compensatory mechanisms could facilitate the earlier identification of disease states.

Some limitations of our study are as follows. First, it was based on participants from one specific hospital in China. Further study is needed to determine how well these findings will generalize to different regions in China (different socio-cultural groups, e.g., urban vs. rural, north vs. south China) and beyond, as well as genetically different (e.g., non-Chinese or non-Asian) populations. Second, our study did not consider medication and the presence of certain diseases; further study is needed to determine whether specific diseases or medications affect the prediction accuracy of the model. Third, we have used the XGBoost machine learning algorithm as a starting point. Future studies could investigate different types of algorithms to determine whether they can better model the relations between blood biomarkers and rRPP.

In conclusion, we have demonstrated that an individual's biochemical/cellular profile predicts resting RPP and have identified important blood biomarkers in this prediction. We have also demonstrated that the XGBoost machine learning algorithm is a good method for modeling such predictions. Future work could determine how well this model generalizes to other populations and disease states, as well as whether accuracy can be further improved. Such models are valuable for understanding the relations between one's biochemical/cellular profile and cardiac workload (rRPP); such an understanding could be helpful for better understanding various physiological and disease states.

## Data Availability

The authors do not have permission to make the data in this study publicly available.
